# Are personnel with a past history of mental disorders disproportionately vulnerable to the effects of deployment-related trauma? A cross-sectional study of Canadian military personnel

**DOI:** 10.1186/s12888-019-2146-z

**Published:** 2019-05-22

**Authors:** Peter J.H. Beliveau, Hugues Sampasa-Kanyinga, Ian Colman, Mark A. Zamorski

**Affiliations:** 1 Directorate of Mental Health, Canadian Forces Health Services Group, 101 Colonel By Drive Carling Campus, Building 9, Ottawa, ON K1A 0K2 Canada; 2 School of Epidemiology, Public Health and Preventive Medicine, University of Ottawa, Ottawa, ON Canada; 3 Department of Family Medicine, University of Ottawa, Ottawa, ON Canada

**Keywords:** Major depressive disorder, Post-traumatic stress disorder, Psychological trauma, Logistic models, Probability, Military personnel

## Abstract

**Background:**

Past mental disorders predict future disorders, both in the presence and absence of trauma exposure. However, it is not clear whether those with past mental disorders are disproportionately vulnerable to the negative effects of a given level of trauma.

**Methods:**

The data source was the 2013 Canadian Forces Mental Health Survey (CFMHS), of which 1820 respondents had deployed only once in their military careers—all in support of the mission in Afghanistan. The primary outcomes were past 12-month depression and past 12-month PTSD. Multivariate logistic regression was performed for each outcome variable, looking primarily for differences in the marginal effect of deployment-related trauma in those with and without a pre-deployment history of each disorder.

**Results:**

A history of each pre-deployment disorder did indeed interact with deployment-related trauma with respect to the corresponding past 12-month disorder. In addition, pre-deployment history of depression and of PTSD interacted with each other, though only for the outcome of past 12-month PTSD. The average marginal effect of deployment-related trauma on past 12-month PTSD was highest in those with a pre-deployment history of depression in the absence of a pre-deployment history of PTSD. This group was twice as vulnerable to post-deployment PTSD relative to those without a pre-deployment history of both disorders and four times as vulnerable to post-deployment PTSD relative to those with a pre-deployment history of both disorders. No significant differences were seen in the marginal effects of trauma on past 12-month depression in the presence or absence of a pre-deployment history of that disorder.

**Conclusion:**

There is modest differential vulnerability to past 12-month PTSD as a function of deployment-related trauma in those who had a pre-deployment history of PTSD or depression when compared to those who did and did not have a pre-deployment history of one or both disorders.

**Electronic supplementary material:**

The online version of this article (10.1186/s12888-019-2146-z) contains supplementary material, which is available to authorized users.

## Background

Psychological trauma exposure is a key determinant of mental health [1–4]. Trauma-related disorders, such as post-traumatic stress disorder, have important impacts on individuals, including poor physical health, poor quality of life, short-term memory loss, and long-term chronic psychological repercussions [5–8]. Mental disorders—whether trauma-related or not—also have impacts on employers, such as productivity loss, absenteeism, medical expenditures, performance problems, and unwanted turnover [9–11].

Military personnel provide a unique opportunity to study associations between psychological trauma exposure and post-traumatic mental health given that they are exposed to isolated periods of high risk exposure to deployment-related trauma while deployed to areas of conflict. Among military personnel a broad range of factors have been shown to increase the risk of developing a mental disorder in the aftermath of deployment-related trauma. Key post-deployment factors include social support [12] and other life stressors [13]. Peri-traumatic factors include those related to the nature of the trauma itself (for example the types of experiences while on deployment) [14] and physical injury due to trauma. Pre-deployment factors include female gender, previous trauma exposure (notably in childhood), and pre-deployment mental health problems [15]. Indeed, a past history of mental disorder is one of the strongest and most consistent predictors of post-traumatic mental health [13, 16, 17].

While there is abundant research on the main effects of pre-deployment *trauma* on post-deployment mental health in military personnel [18–23] and its effect modification on the relationship between deployment-related trauma and mental disorders [19, 24, 25], history of past mental disorder has received less attention with regards to its role in post-deployment mental health. Prior research of PTSD has found that history of witnessing atrocities significantly predicted PTSD after subsequent trauma only among those with prior PTSD and that history of child abuse victimization significantly predicted only among those with no history of prior anxiety disorders [26]. LeardMann et al. [27] have shown that poor mental or physical health prior to combat exposure significantly increased the risk of symptoms or diagnosis of PTSD after deployment. Breslau et al. [28] have suggested that pre-trauma mental health problems could play an important role on the relationship between trauma and subsequent mental health problems in the civilian population. They have shown that antecedent trauma in combination with pre-trauma PTSD was associated with significantly higher risk of PTSD following subsequent trauma, but there was not a significant risk associated with antecedent trauma in the absence of PTSD [28].

A recent study conducted in the Australian military population tested whether pre-deployment mental health modifies the relationship between trauma and post-deployment mental disorder. The results suggested that the relationship between deployment-related trauma and PTSD/depressive symptoms did not significantly differ depending on either pre-deployment trauma experience, prior combat, or pre-deployment PTSD/depressive symptoms [29]. However, this study looked at relatively short-term outcomes among those deployed to Afghanistan across 2010 to 2012, while military organizations are presumably more interested in more persistent cases. In addition, these investigators did not examine whether the different mental disorders interacted [29]. The issue of potential interaction between PTSD and depression is important, given that these disorders commonly occur together and that the conditional risk of persistent or recurrent PTSD (or depression) in this group might be significantly elevated, relative to those with just one of the disorders [30]. If so, this knowledge would inform decision-making regarding continued occupational fitness for personnel with past mental disorder diagnoses who are likely to be exposed to significant deployment-related trauma in their future duties.

The objective of this paper was to explore the marginal effects of pre-deployment mental health disorders with deployment-related trauma exposure in regard to post-deployment disorders. We use survey data from a representative sample of Canadian Armed Forces personnel who deployed in support of the mission in Afghanistan. We hypothesized that for both disorders, the marginal effect of trauma would be greater in those with a pre-deployment history of either disorder and that the effect of trauma on past 12-month mental health problems would be greatest in those with a pre-deployment history of both disorders.

## Methods

### Study population and data source

The 2013 Canadian Forces Mental Health Survey (CFMHS) collected detailed information on mental health problems, their impacts, occupational and non-occupational determinants of mental health, and the use of mental health services [31]. The cross-sectional survey employed a stratified random sampling approach on Canadian Armed Forces (CAF) personnel in service during September 2012. The data were collected by Statistics Canada personnel in mid-2013 via face-to-face, computer-assisted personal interview. The CFMHS had response rates of 79.8 and 78.7% for Regular Forces and Reservists, respectively with a random sample of ~ 8200 serving personnel. However, our analyses are restricted to CAF Regular Force personnel and Reservists who had only ever deployed once prior to data collection and that the deployment was in support of the mission in Afghanistan (population size = 11,860, sample size = 1820). Additional details on the CFMHS are described elsewhere [31].

The relevant ethical and privacy committees within Statistics Canada that serve the functions of a Research Ethics Board reviewed and approved all aspects of data collection (including ethical aspects) and all conditions of data access and use. Participation in the surveys was voluntary, and participants provided informed consent.

### Sequence of events

The events under investigation occurred in the following order: pre-deployment mental disorder, member deployed in support of the mission in Afghanistan, exposure to deployment-related trauma, and identification of post-deployment mental disorder. To ensure that a deployment-related trauma could not have occurred before the pre-deployment mental disorder, participants with more than one deployment were excluded from analyses. Furthermore, prior to deployment all CAF members were required to complete a comprehensive assessment of psychosocial functioning. Pre-deployment assessments were conducted in-person by mental health specialists. The psychometric tests included – but were not limited to: Beck Depression Inventory-II (BDI-II), Patient Health Questionnaire-9 (PHQ-9), Beck Anxiety Inventory (BAI), Generalized Anxiety Disorder 7 (GAD-7), Posttraumatic Stress Disorder Checklist for the DSM-5 (PCL-5), Alcohol Use Disorders Identification Test (AUDIT), Dissociative Experiences Scale (DES or DES Taxon), Outcome Questionnaire (OQ-45.2), and the OTSSC Sleep Apnea Alert. In addition, the mental health history of members was recorded – detailing any contact with primary care clinicians or counselling service providers within the last five years. This mandatory pre-deployment screening was used to identify persistent mental conditions and ensure that those experiencing a mental disorder at the time of screening were not deployed.

### Measures

#### Outcomes: mental disorder

Post-deployment mental disorders that occurred during the preceding 12-month prior to survey data collection including 1) Major Depressive Episode (hereafter “depression”) and 2) PTSD were assessed using the World Health Organization Composite International Diagnostic Interview (WHO-CIDI) under criteria from the Diagnostic and Statistical Manual of the American Psychiatric Association, fourth edition (DSM-IV). The WHO-CIDI is a lay-administered, fully-structured interview that has shown good consistency with clinical diagnostic instruments for the assessment of past 12-month depression and PTSD within the CAF population [32, 33].

#### Independent variable: deployment-related traumatic events

Participants were asked to indicate which of eight possible types of traumatic experiences had ever occurred on deployment. These traumatic experiences were as follows: 1) known someone who was seriously injured or killed; 2) found yourself in a threatening situation where you were unable to respond because of rules of engagement; 3) ever been injured; 4) ever seen ill or injured women or children who you were unable to help; 5) ever received incoming artillery, rocket or mortar fire; 6) ever felt responsible for the death of Canadian or ally personnel; 7) ever had a close call, for example shot or hit but protective gear saved you; and 8) ever had difficulty distinguishing between combatants and non-combatants. These items were selected from a larger inventory of combat experiences developed by the Walter Reed Army Institute for Research [34]. We created a simple sum of the number of these experience types (range, 0 to 8) and treated this as a continuous variable as validated in the literature [35]. Sensitivity analyses showed the same effects when deployment-related traumatic events were treated as categorical variables, grouped into approximate tertiles.

#### Potential modifier: pre-deployment history of mental disorder

Pre-deployment history of depression included any episode of depression prior to deployment and was generated using 1) respondent age at the time of the survey, 2) age of onset of depression, and 3) year of deployment in support of the mission in Afghanistan. The WHO-CIDI instrument collected overall severity, duration, and intensity pertaining to PTSD. However, the version used for the survey did not directly capture the age of onset of PTSD. Therefore, using the approach of other investigators [36], we estimated age of onset of PTSD using year of first traumatic event and the duration between the worst event and first reaction as we did not have a date for the time of first reaction. Pre-deployment history of PTSD was then generated using 1) respondent age, 2) derived age of onset of PTSD, and 3) year of deployment. Sensitivity analyses showed no difference in the thrust of the findings between those who indicated that the first event caused the worst reaction and those in whom the second or subsequent event caused the worst reaction.

Respondent age (in years) and year of deployment were available in the data file. However, the exact date of birth and date of deployment were not. Cases in which onset of mental disorder could not be conclusively determined as pre- or post-deployment were therefore excluded from analysis (1.9% of respondents with lifetime depression, 2.8% with lifetime PTSD) to ensure a clear sequence of pre-trauma disorder, traumatic event exposure, and post-trauma disorder could be established.

#### Covariates: sociodemographic/military characteristics and child abuse victimization

Sociodemographic variables included age (≤ 34, 35 to 44, 45 to 60 years), sex, language of the survey interview (English or French), marital status (married or common law, widowed/separated/divorced, and never married), education (secondary or less, some postsecondary, and postsecondary completion), household income (≤ $59,999, $60,000 to $79,999, and ≥ $80,000), military element (Army, Navy, and Air Force), military component (Regular Force and Reserve Force), rank (junior Non-commissioned member [NCM], senior NCM, and officer), time since return from last Afghanistan deployment (< 1 year, 1–2 years, > 2 years), and childhood victimization. Child victimization was assessed using the Childhood Experiences of Violence Questionnaire [37]. We used the approach of others [2] in collapsing the six source items into any physical abuse, any sexual abuse, and any witnessing of intimate partner violence that had occurred before age 16. We further collapsed these (again, as others have [2]) to a single variable capturing the number of types of child abuse victimization (range, 0–3), which we treated as a scale variable in analyses.

### Statistical analyses

Descriptive analyses were preformed using SAS software, version 9.3. However, regression modeling was done using Stata software, version 14.2. Survey weights were included in the analyses to account for the sample design and to ensure representativeness of the results. Bootstrap methods were used to obtain standard error estimates for descriptive statistics using 500 replicate weights provided by Statistics Canada. All other standard error estimates were calculated using the Taylor Series Linearization method. Missing values were removed by listwise deletion, resulting in the exclusion of cases representing less than 2% of the population. All statistical tests of significance were two-tailed at the α = 0.05 level. Unweighted results are not reported, and weighted estimates were rounded to the nearest 20, in accordance with Statistics Canada’s confidentiality protections.

A series of multivariate logistic regression models were used to examine the relationship between deployment-related trauma and 1) past 12-month depression; and 2) past 12-month PTSD. Model 1 assessed the unadjusted association of deployment-related trauma with the outcomes. Model 2 assessed the association adjusted for sociodemographic and military variables as well as the potential effect modifier of interest (the pre-deployment history of depression or of PTSD), and Model 3 assessed the adjusted association with two-way interactions (using multiplicative interaction terms in the logistic model) between deployment-related trauma and pre-deployment history of mental disorders as well as the interaction between pre-deployment history of depression and pre-deployment history of PTSD. When assessing past 12-month depression, both pre-deployment history of depression and pre-deployment history of PTSD were considered (using a separate variable for each disorder) in Models 2 and 3. An analogous approach was used for the outcome of past 12-month PTSD.

The nature of significant interactions was explored using Stata’s *margins* command, which produced plots of adjusted predicted probabilities as a function of pre-deployment disorder status and deployment-related trauma exposure, as well as estimates of average adjusted marginal effects.

## Results

Of the study population, 9.3% were identified with past 12-month depression and 7.1% with past 12-month PTSD. Respondents were predominantly between the ages of 18 to 44 years, male, and married or in common law relationships (Table 1). The majority served in the Army, with the Regular Force, and were junior non-commissioned members (NCMs). The mean number of deployment-related traumatic events experienced within the sample was 2.89 with a mean of 4.04 and 4.65 events experienced among those with past 12-month depression and PTSD respectively. In total, 91.2% of the sample had returned from deployment two or more years prior to participating in the survey. One or more types of childhood victimization was reported by 47.4% of the sample.

**Table 1:** Demographic and military characteristics of the sample by past 12-month depression and PTSD

Variable	Total sample weighted *N*, %, (95% CI)	Past 12-month depression weighted *N*, %, (95% CI)	Past 12-month PTSD weighted *N*, %, (95% CI)
Total			
Total	11,860, 100%	1100, 9.3% (7.8–10.8)	840, 7.1% (5.8–8.4)
Age‡			
18–34 years	7660, 64.6% (62.0–67.2)	740, 6.2% (5.0–7.5)	520, 4.4% (3.4–5.5)
35–44 years	2580, 21.8% (19.5–24.0)	200, 1.7% (1.1–2.3)	240, 2.0% (1.3–2.8)
45–60 years	1620, 13.7% (12.0–15.4)	160, 1.3% (0.8–1.9)	80, 0.7% (0.3–1.1)
Sex†‡			
Male	10,180, 85.8% (84.0–87.7)	920, 7.8% (6.3–9.2)	680, 5.8% (4.6–7.0)
Female	1680, 14.2% (12.3–16.0)	180, 1.5% (0.8–2.2)	160, 1.4% (0.8–1.9)
Language‡			
English	9220, 77.7% (75.5–80.0)	880, 7.4% (6.1–8.7)	740, 6.3% (5.1–7.5)
French	2640, 22.3% (20.0–24.5)	220, 1.9% (1.1–2.6)	100, 0.9% (0.4–1.3)
Marital status†‡			
Married or common law	7760, 65.4% (63.1–67.8)	620, 5.2% (4.1–6.4)	460, 3.9% (2.9–4.9)
Separated, divorced, or widowed	800, 6.7% (5.4–8.1)	100, 0.8% (0.3–1.4)	80, 0.7% (0.3–1.1)
Never married	3300, 27.8% (25.7–30.0)	380, 3.2% (2.3–4.1)	300, 2.6% (1.7–3.4)
Education†			
High school or less	3960, 33.6% (31.2–36.1)	360, 3.1% (2.2–4.0)	280, 2.4% (1.6–3.2)
Some postsecondary	980, 8.3% (6.9–9.7)	140, 1.2% (0.6–1.7)	60, 0.5% (0.2–0.9)
Post-secondary graduate	6860, 58.2% (55.7–60.8)	620, 5.3% (4.1–6.4)	500, 4.3% (3.3–5.3)
Household income†‡			
< =$60,000	1320, 11.1% (9.7–12.6)	140, 1.2% (0.7–1.7)	120, 1.0% (0.5–1.5)
$60,000–79,999	2920, 24.6% (22.2–27.0)	400, 3.4% (2.4–4.3)	320, 2.7% (1.8–3.6)
> =$80,000	7620, 64.2% (61.8–66.7)	580, 4.9% (3.7–6.0)	400, 3.4% (2.5–4.3)
Service‡			
Army	8980, 75.7% (73.6–77.8)	860, 7.3% (5.9–8.6)	680, 5.8% (4.6–7.0)
Navy	820, 6.9% (5.6–8.2)	80, 0.7% (0.3–1.0)	20, 0.2% (0.0–0.3)
Air Force	2060, 17.4% (15.4–19.4)	160, 1.3% (0.7–2.0)	160, 1.4% (0.8–2.0)
Component			
Regular Force	9480, 79.9% (78.8–81.0)	900, 7.6% (6.1–9.1)	680, 5.8% (4.6–7.0)
Reserve Force	2380, 20.1% (19.0–21.2)	200, 1.7% (1.2–2.1)	160, 1.4% (1.0–1.8)
Rank†‡			
Junior NCM	8000, 67.5% (65.5–69.4)	840, 7.1% (5.7–8.4)	660, 5.6% (4.4–6.8)
Senior NCM	1560, 13.2% (11.7–14.6)	120, 1.0% (0.5–1.5)	80, 0.7% (0.3–1.0)
Officer	2300, 19.4% (17.9–20.9)	160, 1.3% (0.9–1.8)	100, 0.9% (0.4–1.3)
Number deployment-related traumatic events†‡			
0	1500, 12.8% (11.0–14.6)	80, 0.7% (0.3–1.1)	40, 0.3% (0.1–0.6)
1	2020, 17.2% (15.4–19.0)	140, 1.2% (0.6–1.8)	80, 0.7% (0.3–1.1)
2	2060, 17.5% (15.6–19.5)	120, 1.0% (0.4–1.6)	40, 0.3% (0.0–0.6)
3	1800, 15.3% (13.4–17.2)	100, 0.9% (0.4–1.3)	60, 0.5% (0.1–0.9)
4	1680, 14.3% (12.5–16.1)	120, 1.0% (0.5–1.5)	120, 1.0% (0.5–1.6)
5	1260, 10.7% (9.2–12.3)	180, 1.5% (0.9–2.2)	1240, 10.7% (9.1–12.2)
6	800, 6.8% (5.6–8.0)	160, 1.4% (0.8–2.0)	120, 1.0% (0.5–1.6)
7	460, 3.9% (2.9–4.9)	120, 1.0% (0.5–1.5)	140, 1.2% (0.6–1.8)
8	180, 1.5% (0.9–2.1)	80, 0.7% (0.3–1.1)	60, 0.5% (0.2–0.8)
Mean number of traumatic events			
Mean	2.89; SD = 2.01	4.04; SD = 2.37	4.65; SD = 2.17
Time since return from Afghanistan‡			
< 1 year	440, 3.7% (2.7–4.7)	40, 0.3% (0.1–0.6)	20, 0.2% (0.0–0.4)
1–2 years	580, 4.9% (3.6–6.1)	40, 0.3% (0.1–0.6)	40, 0.3% (0.1–0.6)
> 2 years	10,820, 91.2% (89.7–92.8)	1040, 8.8% (7.3–10.2)	800, 6.8% (5.5–8.1)
Number of types of childhood victimization†‡			
0	6240, 52.6% (49.8–55.4)	400, 3.4% (2.4–4.4)	320, 2.7% (1.9–3.6)
1	4140, 34.9% (32.4–37.5)	480, 4.0% (3.1–5.0)	320, 2.7% (1.9–3.6)
2	1300, 11.0% (9.3–12.6)	200, 1.7% (1.1–2.3)	160, 1.4% (0.7–2.0)
3	180, 1.5% (0.8–2.2)	40, 0.3% (0.1–0.6)	40, 0.3% (0.1–0.6)

*PTSD* post-traumatic stress disorder, *CI* confidence interval, *NCM* non-commissioned member, *SD* standard deviation

† Significant at *p*-value < 0.05 when using a Wald chi-square test comparing variable categories for those with past 12-month depression

‡ Significant at *p*-value < 0.05 when using a Wald chi-square test comparing variable categories for those with past 12-month PTSD

History of depression and PTSD prior to deployment is presented in Table 2. Overall, 8.0% of military personnel had a history of depression and 8.3% a history of PTSD prior to deployment. Those with a history of a pre-deployment disorder were much more likely to have the same past 12-month disorder. Of those who reported pre-deployment history of depression, 48.9% were found to have past 12-month depression, versus 5.4% for those without pre-deployment depression. Corresponding prevalence for past 12-month PTSD in those with and without pre-deployment history of PTSD were 20.4% versus 5.9%.

**Table 2:** History of depression and PTSD prior to last deployment in support of the mission in Afghanistan

Variable	Total sample weighted *N*, %, (95% CI)	Past 12-month depression weighted *N*, %, (95% CI)	Past 12-month PTSD weighted *N*, %, (95% CI)
History of depression†‡			
No	10,800, 92.0% (90.5–93.5)	580, 5.4% (4.3–6.6)	680, 6.3% (5.2–7.5)
Yes	940, 8.0% (6.5–9.5)	460, 48.9% (47.9–49.9)	120, 12.8% (12.3–13.4)
History of PTSD†‡			
No	10,880, 91.7% (90.4–93.1)	1000, 9.2% (7.8–10.7)	640, 5.9% (4.7–7.0)
Yes	980, 8.3% (6.9–9.7)	120, 12.2% (11.7–12.7)	200, 20.4% (19.8–21.0)

*CI* confidence interval, *PTSD* post-traumatic stress disorder

† Significant at p-value < 0.05 when using a Wald chi-square test comparing variable categories for those with past 12-month depression

‡ Significant at *p*-value < 0.05 when using a Wald chi-square test comparing variable categories for those with past 12-month PTSD

Logistic regression analyses investigating the association between deployment-related traumatic events and past 12-month depression are presented in Additional file 1: Table S1. Model 1 indicated that deployment-related trauma (treated as a scale variable) was indeed strongly associated with greater odds of past 12-month depression (OR = 1.34, 95% CI [1.23, 1.46]). Results were similar after adjustment for demographic and military characteristics, as presented in Model 2 (OR = 1.46, 95% CI [1.30, 1.64]). Model 3 showed a significant interaction between deployment-related traumatic events and pre-deployment history of depression. However, no statistically significant interaction between a pre-deployment history of depression and pre-deployment a history of PTSD was detected.

### Vulnerability among those with pre-deployment depression

**Fig. 1:**
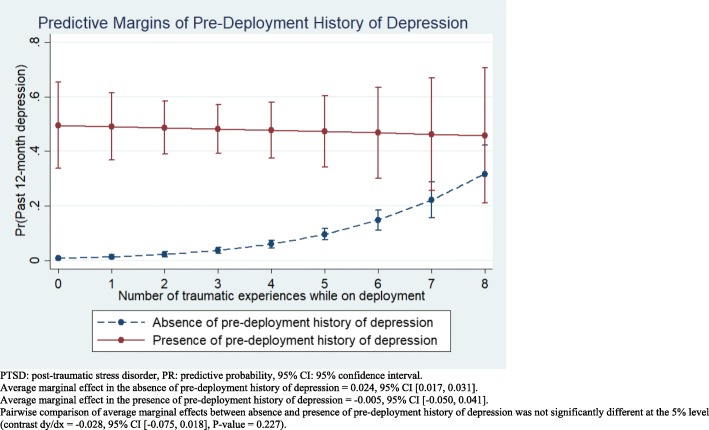
Effect modification by pre-deployment history of depression on the relationship between deployment-related traumatic events and past 12-month depression among Canadian Armed Forces personnel deployed in support of the mission in Afghanistan

The effect modification by pre-deployment history of depression on the relationship between deployment-related traumatic events and past 12-month depression is explored in Fig. 1. A clear main effect of a pre-deployment history of depression is seen across all levels of deployment-related trauma exposure. For example, among those reporting the median number of types of deployment-related trauma (3 types), the adjusted predicted probability, that is the main effect of past 12-month depression in those with and without a pre-deployment history of depression was 48.2% versus 3.7%, respectively (Additional file 3: Table S3). The average marginal effect of deployment-related trauma in those without a pre-deployment history of depression was significantly greater than 0 (average marginal effect = 0.024, 95% CI [0.017, 0.031]), whereas no such relationship was found among those with a pre-deployment history of depression (average marginal effect = − 0.005, 95% CI [− 0.050, 0.041]). However, the difference in average marginal effects in the two groups was not statistically significant, with the wide confidence intervals seen in the group with a pre-deployment history of depression being the apparent explanation. In any case, the marginal effect of trauma in those without a pre-deployment history of depression was small relative to the main effect of a pre-deployment history of depression.

### Vulnerability among those with pre-deployment PTSD

Regression results for past 12-month PTSD (Additional file 2: Table S2) showed a largely similar pattern of findings: There was again a strong relationship between deployment-related trauma and past 12-month PTSD both before and after adjustment for demographic and military characteristics (Model 1, OR = 1.58, 95% CI [1.42, 1.75]; Model 2, OR = 1.71, 95% CI [1.50, 1.94]). The interaction between deployment-related traumatic events and a pre-deployment history of PTSD was also significant, again mirroring the findings for the models using past 12-month depression as the outcome. However, in contrast to the past 12-month depression models, the interaction between pre-deployment PTSD and pre-deployment depression was statistically significant.

**Fig. 2:**
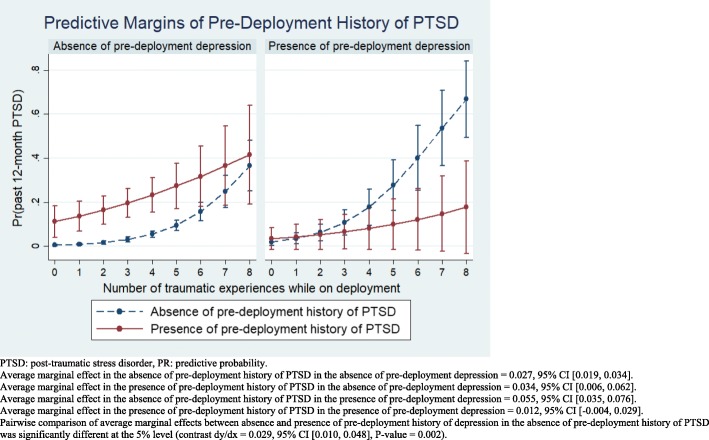
Effect modification by pre-deployment history of PTSD on the relationship between deployment-related traumatic events and past 12-month PTSD with stratified by past 12-month depression among Canadian Armed Forces personnel deployed in support of the mission in Afghanistan

The exploration of the effect modification for past 12-month PTSD is shown in Fig. 2. For this outcome, findings are further stratified by the presence or absence of a pre-deployment history of depression, given the significant interaction detected in the regression models between pre-deployment history of PTSD and pre-deployment history of depression. Sensitivity analyses showed no differences in findings when 3-way interactions were considered. Findings for the marginal effects related to past 12-month PTSD were complex and differed from those involving past 12-month depression. Among those *without* a pre-deployment history of depression, a similar main effect was seen for pre-deployment history of PTSD across all levels of deployment-related trauma exposure, though the difference was not statistically significant for those with the highest deployment-related trauma exposure, for which confidence intervals were broad. The main effect at median level of deployment-related trauma exposure (3 types) was calculated and adjusted predicted probability of past 12-month PTSD in those *with* and *without* a pre-deployment history of PTSD was 19.6% versus 3.1%, respectively (Additional file 4: Table S4).

Of the four stratified groups (those *with*/*without* pre-deployment history of depression and *with/without* pre-deployment history of PTSD) the average marginal effect of deployment-related trauma exposure on past 12-month PTSD was greatest in those *without* a pre-deployment history of PTSD but *with* a pre-deployment history of depression (marginal effect = 0.055, 95% CI [0.035, 0.076]). In contrast, the smallest average marginal effect of deployment-related trauma exposure on past 12-month PTSD was found in those *with* a pre-deployment history of PTSD and *with* a pre-deployment history of depression (marginal effect = 0.012, 95% CI [− 0.004, 0.029]). The intermediate groups—those *with* and *without* a pre-deployment history of PTSD, *without* a pre-deployment history of depression—had similar average marginal effects on past 12-month PTSD (0.034, 95% CI [0.006, 0.062] and 0.027, 95% CI [0.019, 0.034], respectively). Yet, of these four groups defined by the presence/absence of a pre-deployment history of PTSD and the presence/absence of a pre-deployment history of depression, the two pairwise comparison between the average marginal effects of deployment-related trauma that were statistically significant were: 1) that between those *without* a pre-deployment history of PTSD but *with* a pre-deployment history of depression, relative to *without* a pre-deployment history of PTSD and *without* a pre-deployment history of depression, in which a two-fold difference in vulnerability to deployment-related trauma exposure was found (*P*-value < 0.001); and 2) that between those *without* a pre-deployment history of PTSD but *with* a pre-deployment history of depression, relative to *with* a pre-deployment history of PTSD and *with* a pre-deployment history of depression, in which a four-fold difference in vulnerability to deployment-related trauma exposure was found (P-value < 0.001).

## Discussion

### Summary of key findings

In this paper, we explored the differential effects of trauma on past 12-month depression and on past 12-month PTSD in those with and without a pre-deployment history of these mental disorders, specifically any episode of these disorders prior to deployment. We had expected to find a stronger effect of trauma on past 12-month disorders in those with a pre-deployment history of mental disorders, and that this effect would be strongest in those with a pre-deployment history of both disorders. We did indeed find a significant interaction between deployment-related trauma and both a pre-deployment history of depression and a pre-deployment history of PTSD with respect to their respective past 12-month disorders. We also found an interaction between pre-deployment history of depression with pre-deployment history of PTSD for past 12-month PTSD, but not of past 12-month depression.

However, exploring the nature of the significant interactions with adjusted predicted probabilities and average marginal effects yielded a complex picture that did not entirely conform to our hypotheses. For past 12-month depression, deployment-related trauma had a significant effect only in those *without* a pre-deployment history of depression—the opposite of our prediction of a greater effect in those *with* a pre-deployment history of depression. Yet pairwise comparison of the average marginal effect of deployment-related trauma between those *with* and *without* a pre-deployment history of depression was not statistically significant. These seemingly discrepant findings with respect to the marginal effects of deployment-related trauma on past 12-month depression as a function of a pre-deployment history of depression are likely due to the wide confidence intervals seen in the group with a pre-deployment history of depression. The significant interaction term in the regression model is driven by the statistically significant marginal effect of trauma in those without a pre-deployment history of depression and the absence of such a statistically significant difference in those with a pre-deployment history of depression—though the confidence intervals for this estimate were very broad. Those broad confidence intervals likely account for the inability to detect a significant pair-wise difference in the marginal effects of deployment-related trauma in the two groups of interest.

Regardless, it is clear that the difference in marginal effects was small relative to the main effect of a pre-deployment history of depression, with the adjusted predicted probability of past 12-month depression being ten times greater in those with a pre-deployment history of depression relative to those without such a history, at the median level (3 traumatic experiences) of trauma exposure. The frequent relapse and recurrence of depression may explain why the marginal effects we observed were small relative to the main effect of pre-deployment history of depression. In a meta-analysis of 28 studies including 1880 adults it was found that after discontinuation of acute-phase treatment relapse of depression occurred in 29% of the sample within 1 year and 54% with 2 years [38]. Furthermore, 85% of those who recover from depression will experience a second episode within 15 years of naturalistic follow-up with each additional episode increasing risk of recurrence by 18% [39]. Whereas meta-analyses of relapse-recurrence prevalence for PTSD after discontinuation of treatment was found to be 16.4% within 1 year [40].

We did however, have power to detect differences in the marginal effects of deployment-related trauma for past 12-month PTSD. Contrary to our hypothesis, the marginal effect of deployment-related trauma was four times as great in those *without* a pre-deployment history of PTSD and *with* a pre-deployment history of depression relative to those *with* a prior history of both depression and PTSD. The same group had twice the effect when compared to those *without* a prior history of either disorder—a difference that was also statistically significant. The magnitude of the marginal effect was meaningful, representing a four-fold and two-fold greater effect of deployment-related trauma in former group relative to the latter respectively.

Thus, while we confirmed the presence of the interactions we were interested in, the precise nature of the interactions did not conform to our expectations.

### Comparison with other findings

Our results showed that individuals with a history of a pre-deployment disorder were much more likely to have the same past 12-month disorder even after adjusting for important covariates. These findings are consistent with those from previous studies indicating that a history of psychological symptoms prior to exposure to combat related trauma has been associated with the increased likelihood of post-deployment psychiatric conditions including PTSD and mood disorders [41–43].

Searle and colleagues explored the longitudinal relationships among pre-deployment trauma, pre-deployment symptoms of mental disorders, and post-deployment PTSD and depression symptoms. They found an interaction between pre-deployment symptoms of mental disorders and deployment-related trauma, but the interaction disappeared once they controlled for pre-deployment trauma exposure (related to both previous deployments and other life events) [29]. The divergence in the findings may relate to differences in the way their analysis was structured, the use of pre-deployment *symptoms* of mental disorders as opposed to a pre-deployment *history* of mental disorders (our respondents with pre-deployment history of mental disorders may or may not have been symptomatic at the time of deployment), and the timing of assessment of post-deployment mental health problems (months vs. a number of years, on average, in our study). Consistent with the timing of assessment being a potential explanation for the differences in findings, no interaction was seen between combat exposure and past mental health problems in predicting post-deployment mental health symptoms in Canadian Forces personnel 6 months after return from deployment [44]. Other investigators [45–47], however, have found statistically significant (though small) interactions such that those with previous mental health problems had a disproportionate risk of post-deployment mental health symptoms. None of these other studies explored disorder-specific interactions as we did.

### Strengths and limitations

The present study has notable strengths. We used a large, nationally representative sample of serving CAF personnel, many of whom had had significant exposure to deployment-related trauma. We assessed mental disorders using state-of-the art survey assessment tools, and we were able to adjust for a number of confounding factors, including past-child abuse victimization. Finally, we were able to explore disorder-specific interactions with deployment-related trauma from a single isolated experience—an approach that yielded complex and unexpected findings that may explain some of the heterogeneity of past research on this important topic.

Certain limitations must, however, be acknowledged. Most are due to potential problems with respect to the temporality of the events of interest that are typical in cross-sectional studies. First, history of mental disorder was retrospectively reported, thus potentially subject to recall errors [48]. Furthermore, participants were ask to report mental disorders that had been previously diagnosed by a mental health professional. Those with undiagnosed or subclinical mental disorders may have therefore been classified as not having had a pre-deployment mental disorder. Lack of precision of the dates available in the survey data meant that for an important minority the onset of mental disorder was around the time of deployment—though we could not determine if it was before or after; we excluded these individuals in our final analyses. Our dating of the onset of PTSD was subject to some imprecision, as described in the Methods. Nevertheless, we undertook a number of sensitivity analyses to ensure that the imprecision in the assessment of the timing of events did not influence the thrust of the results.

We operationalized pre-trauma and post-trauma disorders to those that occurred prior to or after military deployment. While studying a military population has the advantage of deployment representing a timeframe in which personnel are at high risk of psychological trauma, our results may not hold for other populations. Due to lack of data on timing of non-deployment-related trauma, we were unable to include such events in our analysis. Exposure to non-deployment-related trauma is common among members of the military, and is likely related to both pre- and post-deployment mental health, particularly PTSD [49]. Our assessment and analysis of trauma exposure items had limitations: The eight items used to assess deployment-related trauma represent only a subset of a much larger set of potential events, though the reduced item set we used showed similar psychometric properties to a larger set. In addition, we treated the items analytically as a scale variable—a common and pragmatic approach [50]—though the underlying scaling assumptions may not hold. Nevertheless, treating trauma exposure categorically yielded convergent results in sensitivity analyses (see Methods). Finally, we were only able to control for childhood victimization exposure, but not for other lifetime trauma about which we did not have information about timing relative to deployment.

### Implications

The primary motivation for this analysis was to inform decisions on occupational fitness for personnel with past mental disorder diagnoses who are likely to be exposed to significant deployment-related trauma in their future duties. Occupational health professionals who make these decisions have done so with a firm knowledge of the well-established and well-quantified effects of trauma on mental health and full awareness that those with past disorders are at increased risk for future disorders, whether or not they are exposed to deployment-related trauma. Patients may have a sense of these realities as well.

Less clear has been whether those with past mental health problems are disproportionately vulnerable to the negative effects of deployment-related trauma. As noted above, some studies have demonstrated such an effect whereas others have not—though in no cases have large effects been substantiated. Our findings suggest that those with a pre-deployment history of depression can be reassured with respect to the absence of a significant disproportionate risk of past 12-month depression or of PTSD in the event of future deployment-related trauma. The same is true for those without a pre-deployment history of both PTSD and depression together. For those lacking a pre-deployment history of PTSD and with a pre-deployment history of depression, our findings suggest a two-fold greater vulnerability to deployment-related trauma when it comes to later PTSD (though not, however, with respect to later depression), relative to those without a pre-deployment history of either disorder. Should future exposure to deployment-related trauma for all such patients be precluded on this basis?

For two main reasons, we do not believe so: First, our findings show that many individuals with previous depression do not experience later persistent PTSD: At the median level of trauma exposure (3 event types) more than 90% of those with a pre-deployment history of depression were free of past 12-month PTSD at the time of the survey. Second, the risk of recurrence of PTSD in those with a past history of a mental disorder varies from person-to-person [14, 51, 52], which argues for a more individualized approach to decision-making. To the extent possible, patients should participate in the decision-making, due to the importance of the outcome.

The limitations of our analysis and the uncertainties detailed above highlight the need for future research in this area. Above all else, additional longitudinal analyses with data collection points before and well-after exposure to deployment-related trauma are needed. Assessment of both occupational and non-deployment-related trauma (and their timing) are essential. Finally, research is required to understand individual factors that predict a favorable vs. unfavorable response to future trauma in a given patient with previous mental disorders. Understanding these factors would help inform decision-making on a given patient. Finally, validation of approaches to mitigate excess vulnerability to trauma in those with past disorders is needed.

## Conclusion

Our findings largely provide reassurance with respect to the incremental vulnerability of personnel with past mental health problems to the effects of later deployment-related trauma exposure. While both past disorders and trauma contribute to the risk of later mental health problems, the vulnerability to the effects of trauma on depression and PTSD was similar, with the notable exception that those *without* pre-deployment PTSD and *with* pre-deployment depression were twice as vulnerable with respect to later PTSD, relative to those without any pre-deployment disorders and four times as vulnerable relative to those with a pre-deployment history of both depression and PTSD. While this incremental vulnerability should be taken into account, the magnitude of the differences is modest and hence should not be the sole factor considered when determining occupational fitness. We argue for an individualized approach to such decisions.

## Additional files

Additional file 1: **Table S1.** Associations between deployment-related traumatic events and past 12-month depression in Canadian Armed Forces personnel. (DOCX 21 kb)

Additional file 2: **Table S2.** Associations between deployment-related traumatic events and past 12-month PTSD in Canadian Armed Forces personnel. (DOCX 21 kb)

Additional file 3: **Table S3.** Corresponding margins for Fig. 1 showing effect modification by pre-deployment history of depression on the relationship between deployment-related traumatic events and past 12-month depression among Canadian Armed Forces personnel deployed in support of the mission in Afghanistan. (DOCX 15 kb)

Additional file 4: **Table S4.** Corresponding margins for Fig. 2 showing effect modification by pre-deployment history of PTSD on the relationship between deployment-related traumatic events and past 12-month PTSD stratified by past 12-month depression among Canadian Armed Forces personnel deployed only once in support of the mission in Afghanistan. (DOCX 16 kb)

## Abbreviations

CAF: Canadian Armed Forces
CFMHS: 2013 Canadian Forces Mental Health Survey
DSM-IV: Diagnostic and Statistical Manual of the American Psychiatric Association, Fourth Edition
NCM: Non-commissioned member
PTSD: Post-traumatic stress disorder
WHO-CIDI: World Health Organization Composite International Diagnostic Interview
