# Correction to: Are personnel with a past history of mental disorders disproportionately vulnerable to the effects of deployment-related trauma? A cross-sectional study of Canadian military personnel

**DOI:** 10.1186/s12888-019-2187-3

**Published:** 2019-07-01

**Authors:** Peter J. H. Beliveau, Hugues Sampasa-Kanyinga, Ian Colman, Mark A. Zamorski

**Affiliations:** 1 Directorate of Mental Health, Canadian Forces Health Services Group, 101 Colonel By Drive Carling Campus, Building 9, Ottawa, ON K1A 0K2 Canada; 2 School of Epidemiology, Public Health and Preventive Medicine, University of Ottawa, Ottawa, ON Canada; 3 Department of Family Medicine, University of Ottawa, Ottawa, ON Canada

**Correction to: BMC Psychiatry (2019) 19:156**

**https://doi.org/10.1186/s12888-019-2146-z**

Following publication of the original article [1], we have been notified that the copyright holder needs to be changed from **©The Author(s)** to **©Crown**.

The original article has been corrected.
