# Supplementary material for: Are personnel with a past history of mental disorders disproportionately vulnerable to the effects of deployment-related trauma? A cross-sectional study of Canadian military personnel
Source: BMC Psychiatry. 2019 May 22;19:156. doi: 10.1186/s12888-019-2146-z (PMC6532170; doi:10.1186/s12888-019-2146-z)
Supplement: Supplementary file 1 — Table S1. Associations between deployment-related traumatic events and past 12-month depression in Canadian Armed Forces personnel. (DOCX 21 kb) [file 12888_2019_2146_MOESM1_ESM.docx]

**Supplementary Table S1:** Associations between deployment-related traumatic events and past 12-month depression in Canadian Armed Forces personnel

|  | **Model 1** | | | **Model 2** | | | **Model 3** | | |
| --- | --- | --- | --- | --- | --- | --- | --- | --- | --- |
|  | **OR** | **95% CI** | **P-value** | **OR** | **95% CI** | **P-value** | **OR** | **95% CI** | **P-value** |
| **Number of deployment-related traumatic experiences** | 1.34 | 1.23 - 1.46 | <0.001 | 1.46 | 1.30 – 1.64 | <0.001 | 1.67 | 1.46 – 1.92 | <0.001 |
| **Age** |  |  |  |  |  |  |  |  |  |
| 18-34 years |  |  |  | 1 |  |  |  |  |  |
| 35-44 years |  |  |  | 0.77 | 0.46 – 1.31 | 0.337 | 0.77 | 0.46 – 1.28 | 0.319 |
| 45-60 years |  |  |  | 0.89 | 0.43 - 1.83 | 0.756 | 0.76 | 0.36 – 1.59 | 0.468 |
| **Sex** |  |  |  |  |  |  |  |  |  |
| Male |  |  |  | 1 |  |  |  |  |  |
| Female |  |  |  | 1.51 | 0.88 – 2.58 | 0.133 | 1.33 | 0.74 – 2.38 | 0.338 |
| **Language** |  |  |  |  |  |  |  |  |  |
| English |  |  |  | 1 |  |  |  |  |  |
| French |  |  |  | 1.14 | 0.71 – 1.83 | 0.579 | 1.09 | 0.68 – 1.75 | 0.718 |
| **Marital status** |  |  |  |  |  |  |  |  |  |
| Married or common law |  |  |  | 1 |  |  |  |  |  |
| Separated, divorced, or widowed |  |  |  | 1.76 | 0.69 – 4.48 | 0.238 | 2.16 | 0.95 – 4.94 | 0.067 |
| Never married |  |  |  | 1.29 | 0.82 – 2.01 | 0.269 | 1.39 | 0.89 – 2.15 | 0.145 |
| **Education** |  |  |  |  |  |  |  |  |  |
| High school or less |  |  |  | 1 |  |  |  |  |  |
| Some postsecondary |  |  |  | 1.66 | 0.73 – 3.77 | 0.229 | 1.80 | 0.80 – 4.07 | 0.154 |
| Post-secondary graduate |  |  |  | 1.25 | 0.76 – 2.07 | 0.375 | 1.30 | 0.81 – 2.09 | 0.276 |
| **Household income** |  |  |  |  |  |  |  |  |  |
| <=$60,000 |  |  |  | 1 |  |  |  |  |  |
| $60,000 - 79,999 |  |  |  | 1.66 | 0.82 – 3.34 | 0.157 | 1.46 | 0.75 – 2.84 | 0.262 |
| >=$80,000 |  |  |  | 0.93 | 0.48 – 1.80 | 0.830 | 0.83 | 0.44 – 1.56 | 0.561 |
| **Service** |  |  |  |  |  |  |  |  |  |
| Army |  |  |  | 1 |  |  |  |  |  |
| Navy |  |  |  | 2.71 | 1.27 – 5.77 | 0.010 | 2.77 | 1.24 – 6.16 | 0.013 |
| Air Force |  |  |  | 1.35 | 0.75 – 2.44 | 0.313 | 1.27 | 0.70 – 2.33 | 0.432 |
| **Component** |  |  |  |  |  |  |  |  |  |
| Regular Force |  |  |  | 1 |  |  |  |  |  |
| Reserve Force |  |  |  | 0.91 | 0.58 – 1.41 | 0.670 | 0.83 | 0.54 – 1.28 | 0.410 |
| **Rank** |  |  |  |  |  |  |  |  |  |
| Junior NCM |  |  |  | 1 |  |  |  |  |  |
| Senior NCM |  |  |  | 0.99 | 0.50 – 1.94 | 0.977 | 1.03 | 0.53 – 2.01 | 0.935 |
| Officer |  |  |  | 0.88 | 0.51 – 1.53 | 0.657 | 0.95 | 0.55 – 1.64 | 0.847 |
| **History of depression** |  |  |  |  |  |  |  |  |  |
| No |  |  |  | 1 |  |  |  |  |  |
| Yes |  |  |  | 21.43 | 12.55 – 36.60 | <0.001 | 149.67 | 56.83 – 394.17 | <0.001 |
| **History of PTSD** |  |  |  |  |  |  |  |  |  |
| No |  |  |  | 1 |  |  |  |  |  |
| Yes |  |  |  | 0.78 | 0.36 – 1.69 | 0.527 | 1.26 | 0.59 – 2.68 | 0.552 |
| **Time since return from Afghanistan** |  |  |  |  |  |  |  |  |  |
| <1 year |  |  |  | 1 |  |  |  |  |  |
| 1-2 years |  |  |  | 0.65 | 0.13 – 3.18 | 0.590 | 0.42 | 0.07 – 2.54 | 0.342 |
| >2 years |  |  |  | 1.48 | 0.58 – 3.80 | 0.415 | 1.51 | 0.57 – 3.97 | 0.406 |
| **Number of types of childhood victimization** |  |  |  |  |  |  |  |  |  |
| 0 |  |  |  | 1 |  |  |  |  |  |
| 1 |  |  |  | 1.14 | 0.74 – 1.75 | 0.561 | 1.14 | 0.75 – 1.75 | 0.534 |
| 2 |  |  |  | 1.09 | 0.54 – 2.20 | 0.817 | 1.19 | 0.63 – 2.27 | 0.591 |
| 3 |  |  |  | 1.88 | 0.82 – 4.30 | 0.134 | 2.09 | 0.92 – 4.75 | 0.077 |
| **Number of deployment-related traumatic events X history of depression** |  |  |  |  |  |  | 0.59 | 0.46 – 0.74 | <0.001 |
| **History of depression X history of PTSD** |  |  |  |  |  |  | 0.31 | 0.09 – 1.10 | 0.071 |

PTSD: post-traumatic stress disorder, CI: confidence interval, NCM: non-commissioned member.

Note: No statistically significant three-way interactions (among pre-deployment depression, pre-deployment PTSD, and exposure to deployment-related trauma) were detected for either outcome.
