# Supplementary material for: Are personnel with a past history of mental disorders disproportionately vulnerable to the effects of deployment-related trauma? A cross-sectional study of Canadian military personnel
Source: BMC Psychiatry. 2019 May 22;19:156. doi: 10.1186/s12888-019-2146-z (PMC6532170; doi:10.1186/s12888-019-2146-z)
Supplement: Supplementary file 2 — Table S2. Associations between deployment-related traumatic events and past 12-month PTSD in Canadian Armed Forces personnel. (DOCX 21 kb) [file 12888_2019_2146_MOESM2_ESM.docx]

**Supplementary Table S2:** Associations between deployment-related traumatic events and past 12-month PTSD in Canadian Armed Forces personnel

|  | **Model 1** | | | **Model 2** | | | **Model 3** | | |
| --- | --- | --- | --- | --- | --- | --- | --- | --- | --- |
|  | **OR** | **95% CI** | **P-value** | **OR** | **95% CI** | **P-value** | **OR** | **95% CI** | **P-value** |
| **Number of deployment-related traumatic experiences** | 1.58 | 1.42 – 1.75 | <0.001 | 1.71 | 1.50 – 1.94 | <0.001 | 1.86 | 1.60 – 2.17 | <0.001 |
| **Age** |  |  |  |  |  |  |  |  |  |
| 18-34 years |  |  |  | 1 |  |  |  |  |  |
| 35-44 years |  |  |  | 1.94 | 1.50 – 1.94 | <0.001 | 1.89 | 1.11 – 3.22 | 0.019 |
| 45-60 years |  |  |  | 1.02 | 0.49 – 2.14 | 0.958 | 1.06 | 0.52 – 2.16 | 0.870 |
| **Sex** |  |  |  |  |  |  |  |  |  |
| Male |  |  |  | 1 |  |  |  |  |  |
| Female |  |  |  | 2.44 | 1.34 – 4.46 | 0.004 | 2.06 | 1.11 – 3.83 | 0.023 |
| **Language** |  |  |  |  |  |  |  |  |  |
| English |  |  |  | 1 |  |  |  |  |  |
| French |  |  |  | 0.46 | 0.24 – 0.87 | 0.017 | 0.53 | 0.29 – 0.98 | 0.045 |
| **Marital status** |  |  |  |  |  |  |  |  |  |
| Married or common law |  |  |  | 1 |  |  |  |  |  |
| Separated, divorced, or widowed |  |  |  | 1.08 | 0.41 – 2.86 | 0.872 | 1.16 | 0.48 – 2.81 | 0.749 |
| Never married |  |  |  | 1.67 | 1.01 – 2.74 | 0.044 | 1.65 | 0.99 – 2.75 | 0.053 |
| **Education** |  |  |  |  |  |  |  |  |  |
| High school or less |  |  |  | 1 |  |  |  |  |  |
| Some postsecondary |  |  |  | 1.09 | 0.45 – 2.60 | 0.852 | 1.04 | 0.44 – 2.47 | 0.924 |
| Post-secondary graduate |  |  |  | 1.39 | 0.82 – 2.34 | 0.217 | 1.49 | 0.90 – 2.48 | 0.121 |
| **Household income** |  |  |  |  |  |  |  |  |  |
| <=$60,000 |  |  |  | 1 |  |  |  |  |  |
| $60,000 - 79,999 |  |  |  | 1.18 | 0.59 – 2.36 | 0.648 | 1.23 | 0.62 – 2.46 | 0.557 |
| >=$80,000 |  |  |  | 0.63 | 0.31 – 1.28 | 0.200 | 0.63 | 0.31 – 1.28 | 0.202 |
| **Service** |  |  |  |  |  |  |  |  |  |
| Army |  |  |  | 1 |  |  |  |  |  |
| Navy |  |  |  | 0.46 | 0.15 – 1.39 | 0.168 | 0.40 | 0.13 – 1.23 | 0.110 |
| Air Force |  |  |  | 1.36 | 0.71 – 2.61 | 0.349 | 1.37 | 0.71 – 2.61 | 0.347 |
| **Component** |  |  |  |  |  |  |  |  |  |
| Regular Force |  |  |  | 1 |  |  |  |  |  |
| Reserve Force |  |  |  | 0.75 | 0.47 – 1.19 | 0.227 | 0.76 | 0.48 – 1.20 | 0.236 |
| **Rank** |  |  |  |  |  |  |  |  |  |
| Junior NCM |  |  |  | 1 |  |  |  |  |  |
| Senior NCM |  |  |  | 0.68 | 0.36 – 1.28 | 0.230 | 0.60 | 0.32 – 1.12 | 0.107 |
| Officer |  |  |  | 0.78 | 0.44 – 1.39 | 0.402 | 0.79 | 0.44 – 1.41 | 0.421 |
| **History of depression** |  |  |  |  |  |  |  |  |  |
| No |  |  |  | 1 |  |  |  |  |  |
| Yes |  |  |  | 1.89 | 0.95 – 3.74 | 0.068 | 3.43 | 1.73 – 6.81 | <0.001 |
| **History of PTSD** |  |  |  |  |  |  |  |  |  |
| No |  |  |  | 1 |  |  |  |  |  |
| Yes |  |  |  | 4.45 | 2.53 – 7.81 | <0.001 | 27.37 | 9.97 – 75.09 | <0.001 |
| **Time since return from Afghanistan** |  |  |  |  |  |  |  |  |  |
| <1 year |  |  |  | 1 |  |  |  |  |  |
| 1-2 years |  |  |  | 6.65 | 1.12 – 39.69 | 0.037 | 7.11 | 1.20 – 42.20 | 0.031 |
| >2 years |  |  |  | 3.16 | 0.65 – 15.39 | 0.154 | 3.78 | 0.78 – 18.39 | 0.100 |
| **Number of types of childhood victimization** |  |  |  |  |  |  |  |  |  |
| 0 |  |  |  | 1 |  |  |  |  |  |
| 1 |  |  |  | 1.10 | 0.69 – 1.74 | 0.699 | 1.09 | 0.69 – 1.73 | 0.704 |
| 2 |  |  |  | 1.41 | 0.75 – 2.67 | 0.288 | 1.39 | 0.74 – 2.63 | 0.305 |
| 3 |  |  |  | 2.06 | 0.45 – 9.38 | 0.352 | 1.83 | 0.42 – 8.06 | 0.423 |
| **Number of deployment-related traumatic events X history of PTSD** |  |  |  |  |  |  | 0.68 | 0.54 – 0.86 | 0.001 |
| **History of PTSD X history of depression** |  |  |  |  |  |  | 0.07 | 0.01 – 0.30 | <0.001 |

PTSD: post-traumatic stress disorder, CI: confidence interval, NCM: non-commissioned member.

Note: No statistically significant three-way interactions (among pre-deployment depression, pre-deployment PTSD, and exposure to deployment-related trauma) were detected for either outcome.
