# Supplementary material for: Are personnel with a past history of mental disorders disproportionately vulnerable to the effects of deployment-related trauma? A cross-sectional study of Canadian military personnel
Source: BMC Psychiatry. 2019 May 22;19:156. doi: 10.1186/s12888-019-2146-z (PMC6532170; doi:10.1186/s12888-019-2146-z)
Supplement: Supplementary file 3 — Table S3. Corresponding margins for Fig. 1 showing effect modification by pre-deployment history of depression on the relationship between deployment-related traumatic events and past 12-month depression among Canadian Armed Forces personnel deployed in support of the mission in Afghanistan. (DOCX 15 kb) [file 12888_2019_2146_MOESM3_ESM.docx]

**Supplementary Table S3:** Corresponding margins for Figure 1 showing effect modification by pre-deployment history of depression on the relationship between deployment-related traumatic events and past 12-month depression among Canadian Armed Forces personnel deployed in support of the mission in Afghanistan

| **Number of deployment-related traumatic experiences** | **Absence of pre-deployment depression** | | | **Presence of pre-deployment depression** | | |
| --- | --- | --- | --- | --- | --- | --- |
|  | **Margin** | **95% CI** | **P-value** | **Margin** | **95% CI** | **P-value** |
| **0** | 0.008 | 0.003 – 0.014 | 0.003 | 0.496 | 0.338 – 0.653 | <0.001 |
| **1** | 0.014 | 0.006 – 0.021 | <0.001 | 0.491 | 0.368 – 0.614 | <0.001 |
| **2** | 0.023 | 0.013 – 0.032 | <0.001 | 0.486 | 0.390 – 0.583 | <0.001 |
| **3** | 0.037 | 0.026 – 0.489 | <0.001 | 0.482 | 0.393 – 0.570 | <0.001 |
| **4** | 0.061 | 0.046 – 0.075 | <0.001 | 0.477 | 0.375 – 0.579 | <0.001 |
| **5** | 0.096 | 0.076 – 0.117 | <0.001 | 0.472 | 0.342 – 0.603 | <0.001 |
| **6** | 0.149 | 0.112 – 0.186 | <0.001 | 0.468 | 0.301 – 0.634 | <0.001 |
| **7** | 0.222 | 0.156 – 0.288 | <0.001 | 0.463 | 0.257 – 0.669 | <0.001 |
| **8** | 0.317 | 0.211 – 0.423 | <0.001 | 0.458 | 0.211 – 0.706 | <0.001 |

95% CI: 95% confidence interval.
