# Supplementary material for: Are personnel with a past history of mental disorders disproportionately vulnerable to the effects of deployment-related trauma? A cross-sectional study of Canadian military personnel
Source: BMC Psychiatry. 2019 May 22;19:156. doi: 10.1186/s12888-019-2146-z (PMC6532170; doi:10.1186/s12888-019-2146-z)
Supplement: Supplementary file 4 — Table S4. Corresponding margins for Fig. 2 showing effect modification by pre-deployment history of PTSD on the relationship between deployment-related traumatic events and past 12-month PTSD stratified by past 12-month depression among Canadian Armed Forces personnel deployed only once in support of the mission in Afghanistan. (DOCX 16 kb) [file 12888_2019_2146_MOESM4_ESM.docx]

**Supplementary Table S4:** Corresponding margins for Figure 2 showing effect modification by pre-deployment history of PTSD on the relationship between deployment-related traumatic events and past 12-month PTSD stratified by past 12-month depression among Canadian Armed Forces personnel deployed only once in support of the mission in Afghanistan

| **Number of deployment-related traumatic experiences** | **Absence of pre-deployment Depression** | | | | | | **Presence of pre-deployment Depression** | | | | | |
| --- | --- | --- | --- | --- | --- | --- | --- | --- | --- | --- | --- | --- |
|  | **Absence of pre-deployment PTSD** | | | **Presence of pre-deployment PTSD** | | | **Absence of pre-deployment PTSD** | | | **Presence of pre-deployment PTSD** | | |
|  | **Margin** | **95% CI** | **P-value** | **Margin** | **95% CI** | **P-value** | **Margin** | **95% CI** | **P-value** | **Margin** | **95% CI** | **P-value** |
| **0** | 0.005 | 0.001 – 0.009 | 0.009 | 0.112 | 0.040 – 0.184 | 0.002 | 0.019 | 0.004 – 0.035 | 0.016 | 0.034 | -0.015 – 0.084 | 0.178 |
| **1** | 0.009 | 0.004 – 0.015 | 0.001 | 0.136 | 0.068 – 0.204 | <0.001 | 0.035 | 0.010 – 0.060 | 0.005 | 0.043 | -0.0150 – 0.100 | 0.147 |
| **2** | 0.017 | 0.009 – 0.248 | <0.001 | 0.164 | 0.010 – 0.229 | <0.001 | 0.062 | 0.025 – 0.100 | 0.001 | 0.053 | -0.014 – 0.120 | 0.123 |
| **3** | 0.031 | 0.020 – 0.041 | <0.001 | 0.196 | 0.131 – 0.262 | <0.001 | 0.108 | 0.051 – 0.164 | <0.001 | 0.066 | -0.013 – 0.145 | 0.105 |
| **4** | 0.054 | 0.040 – 0.069 | <0.001 | 0.233 | 0.155 – 0.310 | <0.001 | 0.178 | 0.095 – 0.260 | <0.001 | 0.081 | -0.014 – 0.176 | 0.094 |
| **5** | 0.095 | 0.072 – 0.117 | <0.001 | 0.273 | 0.171 – 0.376 | <0.001 | 0.276 | 0.161 – 0.391 | <0.001 | 0.100 | -0.015 – 0.215 | 0.089 |
| **6** | 0.157 | 0.117 – 0.199 | <0.001 | 0.318 | 0.180 – 0.455 | <0.001 | 0.400 | 0.252 – 0.548 | <0.001 | 0.122 | -0.018 – 0.262 | 0.089 |
| **7** | 0.248 | 0.175 – 0.322 | <0.001 | 0.365 | 0.185 – 0.545 | <0.001 | 0.537 | 0.366 – 0.707 | <0.001 | 0.148 | -0.024 – 0.319 | 0.092 |
| **8** | 0.366 | 0.251 – 0.481 | <0.001 | 0.415 | 0.190 – 0.640 | <0.001 | 0.668 | 0.494 – 0.841 | <0.001 | 0.178 | -0.032 – 0.388 | 0.097 |

PTSD: post-traumatic stress disorder, 95% CI: 95% confidence interval.
